# Supplementary material for: The Systems Biology Research Tool: evolvable open-source software
Source: BMC Syst Biol. 2008 Jun 29;2:55. doi: 10.1186/1752-0509-2-55 (PMC2446383; doi:10.1186/1752-0509-2-55)
Supplement: Additional file 1 — SBRT Archive. An archive of the current version of the Systems Biology Research Tool. [file 1752-0509-2-55-S1.zip › sbrt-1.4.0/doc/developers_guide/api/sbrt/shell/proc/fba/BiggSbmlTranslater.html]

BiggSbmlTranslater


|  |  |  |
| --- | --- | --- |
| **Overview** \| **Package** \| **Class** \| **Use** \| **Tree** \| **Deprecated** \| **Index** \| **Help** | |  |
| **PREV CLASS**   **NEXT CLASS** | **FRAMES**    **NO FRAMES**     **All Classes** |  |
| SUMMARY: NESTED \| FIELD \| CONSTR \| METHOD | DETAIL: FIELD \| CONSTR \| METHOD |  |


---


## sbrt.shell.proc.fba Class BiggSbmlTranslater

```
java.lang.Object
  sbrt.shell.proc.fba.BiggSbmlTranslater
```

---

``` public class BiggSbmlTranslater extends java.lang.Object ```

This class is used to translate SBML files from the BiGG Database into the
Systems Biology Research Tool's *FBA Reaction Files* and *FBA
Catalyst Files*.

**Author:**
:   This class was written and documented by Jeremiah Wright while in the
    Wagner lab.

---

**Constructor Summary**

`BiggSbmlTranslater()`
  
          Constructs a new SBML translater.


| **Method Summary** | |
| --- | --- |
| `void` | `translate(java.lang.String sbmlFileName, java.lang.String rxnFileName, java.lang.String geneFileName, java.io.PrintWriter out)`             Translates an SBML file into an FBA Reaction File and an FBA Catalyst File. |

**Methods inherited from class java.lang.Object**

`clone, equals, finalize, getClass, hashCode, notify, notifyAll, toString, wait, wait, wait`

**Constructor Detail**

### BiggSbmlTranslater

```
public BiggSbmlTranslater()
```

:   Constructs a new SBML translater.


**Method Detail**

### translate

```
public void translate(java.lang.String sbmlFileName,
                      java.lang.String rxnFileName,
                      java.lang.String geneFileName,
                      java.io.PrintWriter out)
```

:   Translates an SBML file into an FBA Reaction File and an FBA Catalyst
    File.

    :   **Parameters:**: `sbmlFileName` - the name of the SBML file.: `rxnFileName` - the desired name of the FBA Reaction File.: `geneFileName` - the desired name of the FBA Catalyst File to which genes will be written.: `out` - the print writer to which descriptive messages will be written.


---


|  |  |  |
| --- | --- | --- |
| **Overview** \| **Package** \| **Class** \| **Use** \| **Tree** \| **Deprecated** \| **Index** \| **Help** | |  |
| **PREV CLASS**   **NEXT CLASS** | **FRAMES**    **NO FRAMES**     **All Classes** |  |
| SUMMARY: NESTED \| FIELD \| CONSTR \| METHOD | DETAIL: FIELD \| CONSTR \| METHOD |  |


---
